# Temporal and spatial variations of soil C, N contents and C:N stoichiometry in the major grain-producing region of the North China Plain

**DOI:** 10.1371/journal.pone.0253160

**Published:** 2021-06-11

**Authors:** Huan Yang, Xuan Song, Yun Zhao, Weitong Wang, Zhennan Cheng, Qi Zhang, Daoquan Cheng

**Affiliations:** 1 School of Water Conservancy Engineering, Zhengzhou University, Zhengzhou, Henan, China; 2 School of Software, Zhengzhou University, Zhengzhou, Henan, China; 3 Station of Soil and Fertilizer Extension Service, Zhengzhou, Henan, China; Shandong University, CHINA

## Abstract

Soil C, N contents and C:N stoichiometry are important indicators of soil quality, the variation characteristics of which have great significance for soil carbon-nitrogen cycle and sustainable utilization. Based on 597 observations along with soil profiles of 0–20cm depth in the 1980s and the 2010s, the temporal and spatial variations of soil C, N contents and C:N stoichiometry in the major grain-producing region of the North China Plain were illustrated. Results showed that there were significant changes in soil C, N contents over time, with increasing rates of 60.47% and 50%, respectively. The changes of C, N contents resulting in a general improvement of C:N stoichiometry. There was a significant decline in nugget effects of soil C, N contents from the 1980s to 2010s, the spatial autocorrelation of soil nutrients showed an increasing trend, and the effect of random variation was reduced. C:N stoichiometry was higher in Huixian City and Weihui City, and lower in Yanjin County, an apparent decline was observed in the spatial difference of soil C:N stoichiometry from the 1980s to 2010s. Soil C, N contents and C:N stoichiometry differed among soil types, agricultural land-use types, and topography in space. The temperature, precipitation, and fertilization structure were considered as the main factors that induce the temporal variations. These findings indicated that the soil nutrient elements in the farmland ecosystems changed in varying degrees in both time and space scales, and the variation was influenced by soil types, land-use types, topography, meteorological factors, and fertilization structure.

## Introduction

Soil organic carbon(C) and nitrogen(N), the basic nutrient elements for plant growth and development, are not only the core of soil nutrient cycling and transformation but also important parts of terrestrial soil C and N storage [1,2]. The changes in soil C, N contents and their ratios are considered to be especially important because they are closely related to the evolution processes of soil quality and fertility, and directly affect soil nutrient status [3–5]. Ecological stoichiometry considers the dynamic balance and coupling relationship of elements in ecological interactions by combining the basic theories of biology, chemistry, physics, and ecology [6], it is an important tool for elaborating ecological processes such as energy flow, material circulation, and nutrient restriction in ecosystems [7]. Thus, exploring the ecological stoichiometry of soil C, N can help to improve our understanding of the soil nutrient characteristics under the synthetic effect of environmental factors, which is of great significance for illustrating the spatial and temporal variations of soil nutrient elements [8–10].

For the past few years, there has been an increasing body of knowledge dedicated to the field of ecological stoichiometry [11–15]. The existing researches have elucidated the specific patterns of C, N contents and C:N stoichiometry in ecosystems with typical biological and ecological features, such as forests, wetlands, grasslands, and coastal estuary [16–19], but little attention has been paid to the ecological stoichiometry of soil C, N in farmland ecosystems. Compared with natural soil, C, N in cultivated soil are more active in the global carbon and nitrogen storage, meanwhile, they are vulnerable to the change of environmental factors, such as climate change, land-use patterns, and agricultural management practices [20]. China is an agricultural country with a large proportion of arable land, over the past couple of decades, the North China Plain has always been regarded as one of the most productive agricultural regions of China [21,22]. However, as far as we know, researches about the ecological stoichiometry of soil nutrient elements in farmland ecosystems are little documented.

In the present study, we aimed to investigate the variations of soil C, N contents and C:N stoichiometry in the major grain-producing region of the North China Plain in both time and space scales, and explore the possible influencing factors and their effects on the temporal and spatial variations of soil C, N contents and C:N ratio in farmland ecosystems.

## Materials and methods

### Study area

**Fig 1:**
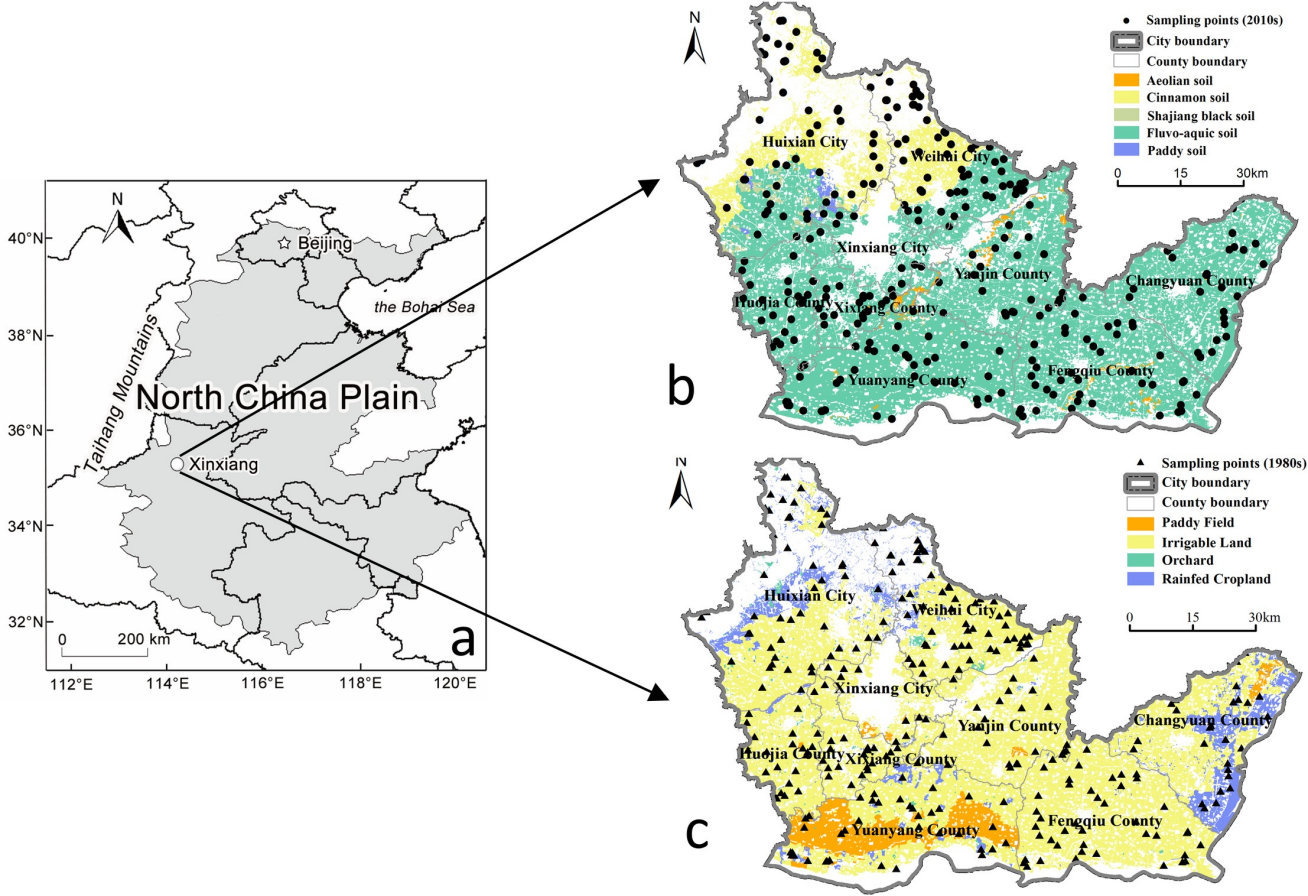
The study area and distribution of sampling sites. a. Location of the study area. b. Distribution of sampling sites in the 1980s and soil types. c. Distribution of sampling sites in the 2010s and agricultural land-use types.

As one of the major grain-producing regions of North China Plain, Xinxiang is located at the southwest of the North China Plain (113°30′-115°30′E, 34°55′-35°50′N) (Fig 1A), with a cultivable land area of about 5062 km^2^, accounting for 61.36% of the total area of the region. It belongs to the warm temperate continental monsoon climate zone, with an annual average temperature of 14°C and average annual precipitation of 573.4 mm. The study area borders the Taihang Mountain to the north and the Yellow River to the south, along with the decrease of elevation, the terrain alters from hilly areas to the plain. Complicated topographic features lead to a diversity of soil types (Fig 1B) and land-use types (Fig 1C), which made Xinxiang a typical agricultural region in North China Plain. Wheat and maize are the main crops in the study area, there is also a small amount of orchard, rape, and oil crops.

### Data source and processing

Soil profile data in the study area was from the second soil survey of Henan province (in the 1980s) and the cultivated land productivity evaluation program that was carried in the 2010s (S1 Table). The locations, sampling depth, parent materials, soil types, soil C content, soil N content, land use patterns, and other related information of each sample site in the 1980s and the 2010s were well recorded in thematic paper documents [23,24]. In the sampling process, 1 kg of soil sample at 0–20 cm depth was collected by using the multi-point mixed sampling method. After natural air-drying, soil samples were ground and screened in the laboratory. C content was determined by the potassium dichromate external heating method, N content was determined by the Kjeldahl method. In this study, C, N contents refer to C and N along with soil profiles of 0–20 cm depth, C:N stoichiometry refers to the mass fraction ratio of C to N. In this study, ArcGIS 10.2 was used to digitize and vectorize the soil profile data, display the spatial distribution of soil C, N contents and C:N stoichiometry, as well as providing the basis for spatial analysis.

The meteorological data used in this study was from China Meteorological Data Service Center (CMDC) (http://data.cma.cn/en). Fertilizer application data was collected from "Statistical Yearbooks"(http://tjj.xinxiang.gov.cn/) and other related agricultural statistics.

(Ethics Statement: The soil profile data was from the second soil survey of Henan province and the cultivated land productivity evaluation program. Permissions were obtained from the administrative department to allow soil sampling. The soil samples are topsoil, so there is no effect on the surrounding environment. There is no endangered or protected species involved in the research.)

### Methodology

Due to the interaction between space phenomena in different directions and distances, traditional mathematical statistics methods are not effective to deal with the problems of spatial valuation and topological relation of spatial objects [25]. In this study, the semi-variogram and the ordinary Kriging method were used to describe the characteristics of C, N contents and C:N ratio in different periods with the help of GS+(v9.0) and ArcGIS 10.2.

Semi-variogram is a function about the relationship between semi-variance and distance of data points, it describes the spatial structural changes, as well as the random changes of regionalized variables, the calculation formula of the semi-variogram is as follows [26]:

$$\gamma (h)=\frac{1}{2N(h)}\sum_{i=1}^{N(h)}{\left[Z(X_{i})-z(X_{i}+h)\right]}^{2}$$ (1)

where: γ(h) is semi-variogram; h is the distance between sample sites; N(h) is the number of pairs with the separation lag h; Z(X_i_) and z(X_i_+h) are observations at spatial positions of X_i_ and X_i_+h, respectively.

The ordinary Kriging method, one of the most commonly used spatial interpolation methods, was used for the spatial analysis of soil C, N contents and C:N ratio. The method can estimate values of environmental variables at unsampled locations with existing sample data and semi-variance function, it is also able to calculate the error of each estimated value, thus the reliability of the estimated results could be significantly improved. The calculation formula of the ordinary Kriging method is as follows [27]:

$$\hat{z}(S_{0})=\sum_{i=1}^{n}W_{i}∙z(S_{i})$$ (2)

where: z(S_i_) is the attribute value of i th measured location; W_i_ is the weight of the measured value of i th location; S_0_ is the point to be interpolated; n is the number of sample sites.

K-S method was used to test whether all sample data were distributed normally in SPSS 21.0. The differences between the variables in different statistical dimensions were evaluated by one-way ANOVA, and origin 2017 was used for data visualization.

## Results

### Descriptive statistics of soil C, N contents and C:N stoichiometry

From the 1980s to the 2010s, soil C content in the study area increased from 5.97 g•kg^-1^ to 9.58 g•kg^-1^, with an increased rate of 60.47%. Soil N content increased from 0.68 g•kg^-1^ to 1.02 g•kg^-1^, with an increase rate of 50.00%. Different variation degrees of C, N contents induced change of C:N ratio. During the past 30 years, C:N ratio increased from 8.95 to 9.37 (Table 1). There was a significant improvement in soil nutrient condition, but the contents of soil C and N were still 30.33% and 28.17% below the national average level (C:13.75 g•kg^-1^, N:1.42 g•kg^-1^). In terms of the coefficient of variation, C, N contents and C:N ratio showed moderate variability with variation coefficients of 59.12%, 52.94% and 27.82%, respectively in the 1980s. In the 2010s, C, N contents still showed moderate variability with variation coefficients of 30.16%, 28.43%, respectively, while the variation degree of the C:N ratio changed from moderate variability to low variability. It indicates that the soil C:N ratio in the study area tends to distribute evenly in space over the past 30 years.

**Table 1:** Descriptive statistics of C, N contents and C:N ratio.

Variable	Period	Min(g·kg^-1^)	Max(g·kg^-1^)	Mean*(g·kg^-1^)	SD	CV(%)
C	1980s	0.65	20.42	5.97±0.21	3.53	59.12
	2010s	4.19	21.43	9.58±0.17	2.89	30.16
N	1980s	0.05	2.24	0.68±0.02	0.36	52.94
	2010s	0.47	2.21	1.02±0.02	0.29	28.43
C:N	1980s	1.52	22.94	8.95±0.15	2.49	27.82
	2010s	4.72	13.21	9.37±0.04	0.64	6.83

### Geostatistical characteristics of C, N contents and C:N stoichiometry

The geostatistical analysis was performed by GS+ (v9.0), the values of key parameters of semi-variogram and the best-fitting models were shown in Table 2. *C*_0_ is the nugget, representing the spatial variation induced by random factors. *C*_0_+*C* is the sill, representing the total variation degree of spatial variables. C0(C0+C) is the nugget effect, which reflects the proportion of spatial variation induced by random factors to the total variation of the system. In general, when C0(C0+C)<25%, it indicates that the variables have a strong spatial correlation, when 25%<C0(C0+C)<75%, it indicates that the variables have a moderate spatial correlation, when C0(C0+C)>75%, it indicates that the spatial correlation of variables is weak. The optimal spatial model was selected according to the larger R^2^ and the smaller RSS. Table 2 suggested that the best fitting models of C, N contents and C:N ratio in the 1980s were the exponential model, gaussian model, and spherical model. In the 2010s, the exponential model was the optimal model for soil C content and C:N ratio, and the optimal fitting model for soil N content was the spherical model. C, N contents and C:N ratio had moderate spatial autocorrelation in the 1980s with nugget effects of 31.23%, 36.68% and 49.76%, respectively. While in the 2010s, C, N contents showed strong spatial autocorrelation with nugget effects of 0.46% and 1.36%, respectively. It indicated that the spatial autocorrelation of soil C, N contents showed a significant increasing trend from the 1980s to 2010s, and the effect of random variation was reduced in the major grain-producing region.

**Table 2:** Parameters of soil C, N contents and C:N ratio in semi-variogram model.

Period	Variable	Model	C_0_	C_0_+C	C_0_/(C_0_+C)	A(km)	RSS	R^2^
1980s	C	Exponential	0.1381	0.4422	31.23%	39.0	4.55E-03	0.943
	N	Gaussian	0.0234	0.0638	36.68%	70.3	5.36E-05	0.945
	C:N	Spherical	0.1056	0.2122	49.76%	31.6	2.45E-03	0.819
2010s	C	Exponential	0.0400	8.6460	0.46%	50.0	2.56	0.976
	N	Spherical	0.0012	0.0883	1.36%	47.4	2.67E-04	0.975
	C:N	Exponential	0.2032	0.4074	49.88%	32.1	0.0131	0.655

### Temporal and spatial variations of C, N contents and C:N stoichiometry

**Fig 2:**
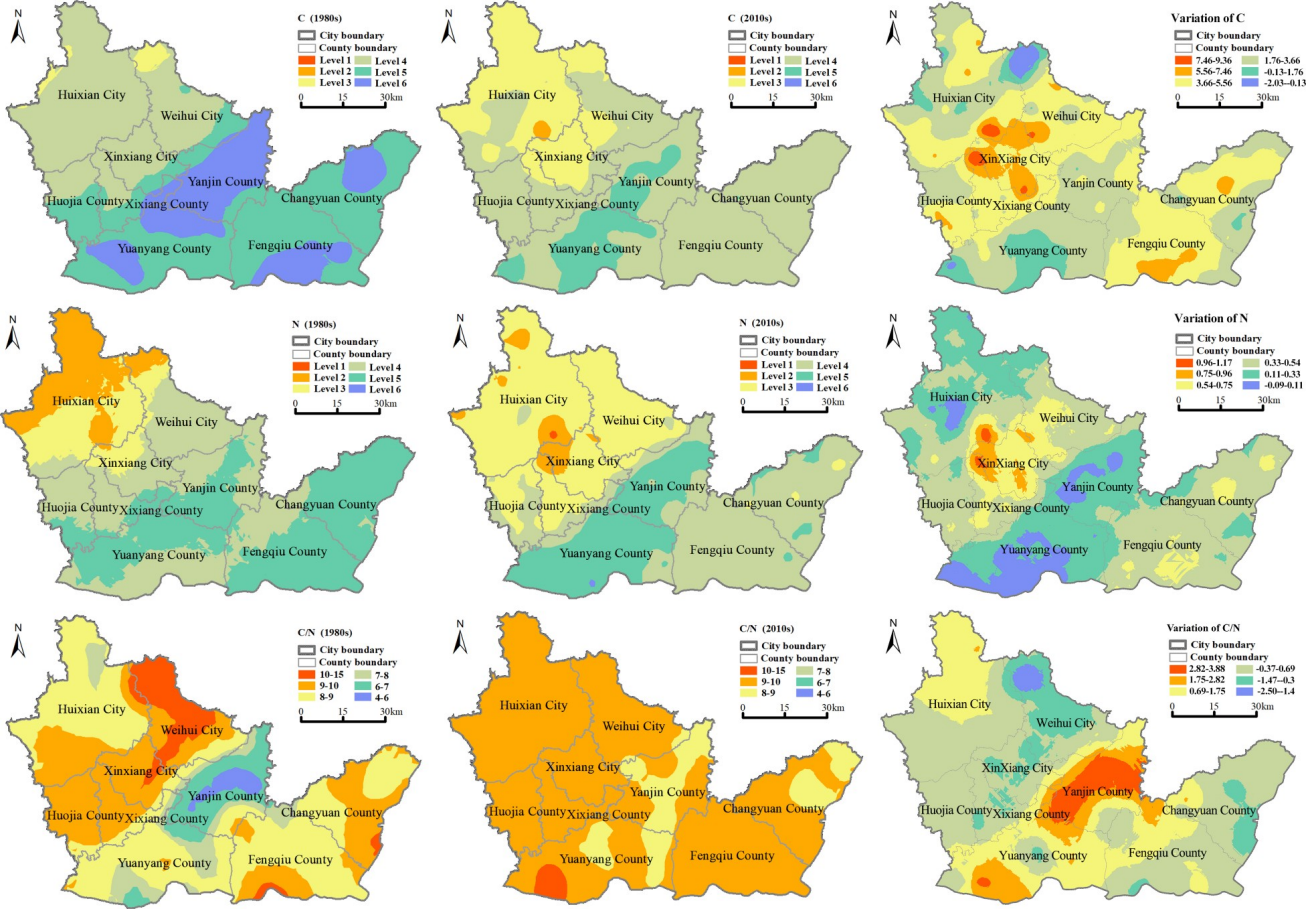
Spatial distribution and variation of C, N contents and C:N stoichiometry in the 1980s and the 2010s.

**Fig 3:**
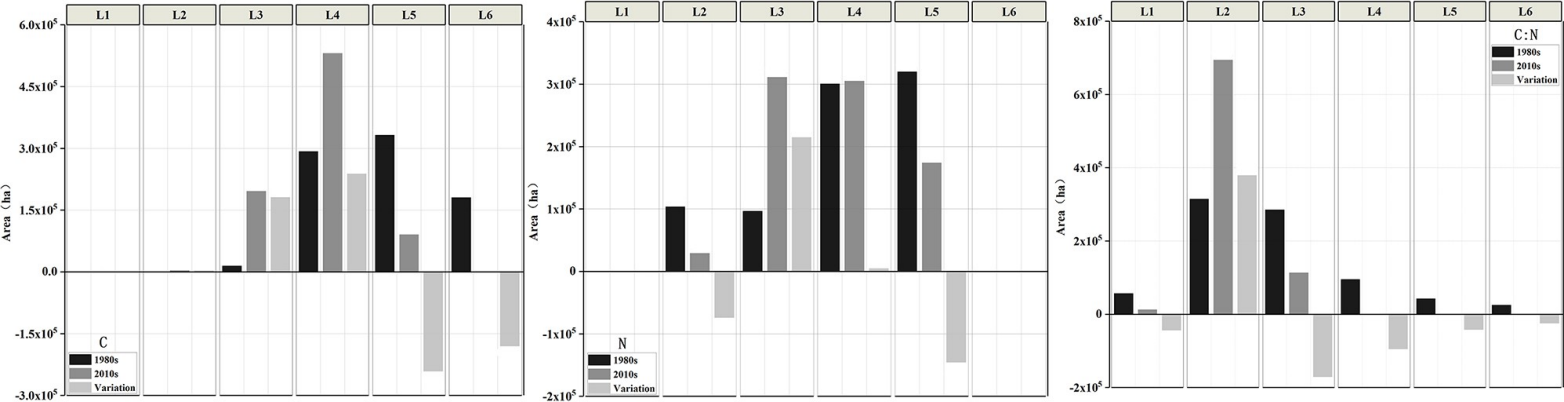
Area of C, N contents and C:N stoichiometry at each level.

Soil C, N contents were divided into six levels from level 1 to level 6 according to the national classification standard for soil nutrient content [28] (Table 3). According to the K-S test, soil C, N contents and C:N ratio in the 1980s and the 2010s were accords with the normal distribution. Based on the optimal semi-variogram models, the ordinary Kriging method was used to predict the spatial distribution of soil nutrient elements in the study area. Fig 2 shows the variation of soil C, N contents and C:N ratio from the 1980s to the 2010s in the major grain-producing regions of North China Plain, and the area of different levels of C, N contents and C:N ratio was calculated by using the spatial analysis function of ArcGIS 10.2 (Fig 3).

**Table 3:** National classification standard for soil nutrient content (part).

	Level 1	Level 2	Level 3	Level 4	Level 5	Level 6
SOC (%)	>4.00	3.01–4.00	2.01–3.00	1.01–2.00	0.60–1.00	<0.60
TN (%)	>0.200	0.151–0.200	0.100–0.150	0.076–0.100	0.050–0.076	<0.050

The northwest of Huixian City and the northern of Weihui City had high values of C content, the low-value areas were mainly distributed in Yanjin County, Yuanyang County, and Fengqiu County in both periods. In the past 30 years, the area of C content within the range of level 4 showed the biggest growth with a value of 238179 ha, followed by level 3 and level 2, which were 181202 ha and 3490 ha. The greatest growth of C content was observed at the intersection of Xinxiang City and Weihui City, with an average growth of 5.56 g•kg^-1^, while C content of the central of Yuanyang County and the northwest of Huixian City was decreased with an average reduction of 2.03 g•kg^-1^.

In the 1980s, high N content was appeared in Huixian City, while in the 2010s, the area with high-value of N content changed to the middle of the study area, such as Xinxiang City and the southeast region of Huixian City. From the 1980s to the 2010s, the area of N content within the range of level 3 increased 214707 ha, followed by level 4 and level 1, the growths of which were 4556 ha and 621 ha, respectively. The region with increased N content was mainly distributed in the junction of Xinxiang County and Huixian City, while the decreased trend of N content was observed in Yuanyang County, Yanjin County, and Huixian County.

C:N ratio in the 1980s showed an increasing trend from the center to periphery with values ranged from 4 to 15. The high-value area of C:N ratio was distributed in Weihui City, and the low-value area was mainly in Yanjin County. In the 2010s, soil C:N ratio was between 9 and 10. The south of Yuanyang country had a higher C:N ratio, and there was no conspicuous low-value region. It indicated that there was a significant decrease in the spatial difference of soil C:N ratio, the balance of soil nutrient condition has been generally improved. From the 1980s to the 2010s, the soil C:N ratio has increased in most regions of the study area, this trend is beneficial to the accumulation of soil organic carbon and the sustainable development of agriculture.

## Discussion

### Effects of soil types on soil C, N contents and C:N ratio

**Fig 4:**
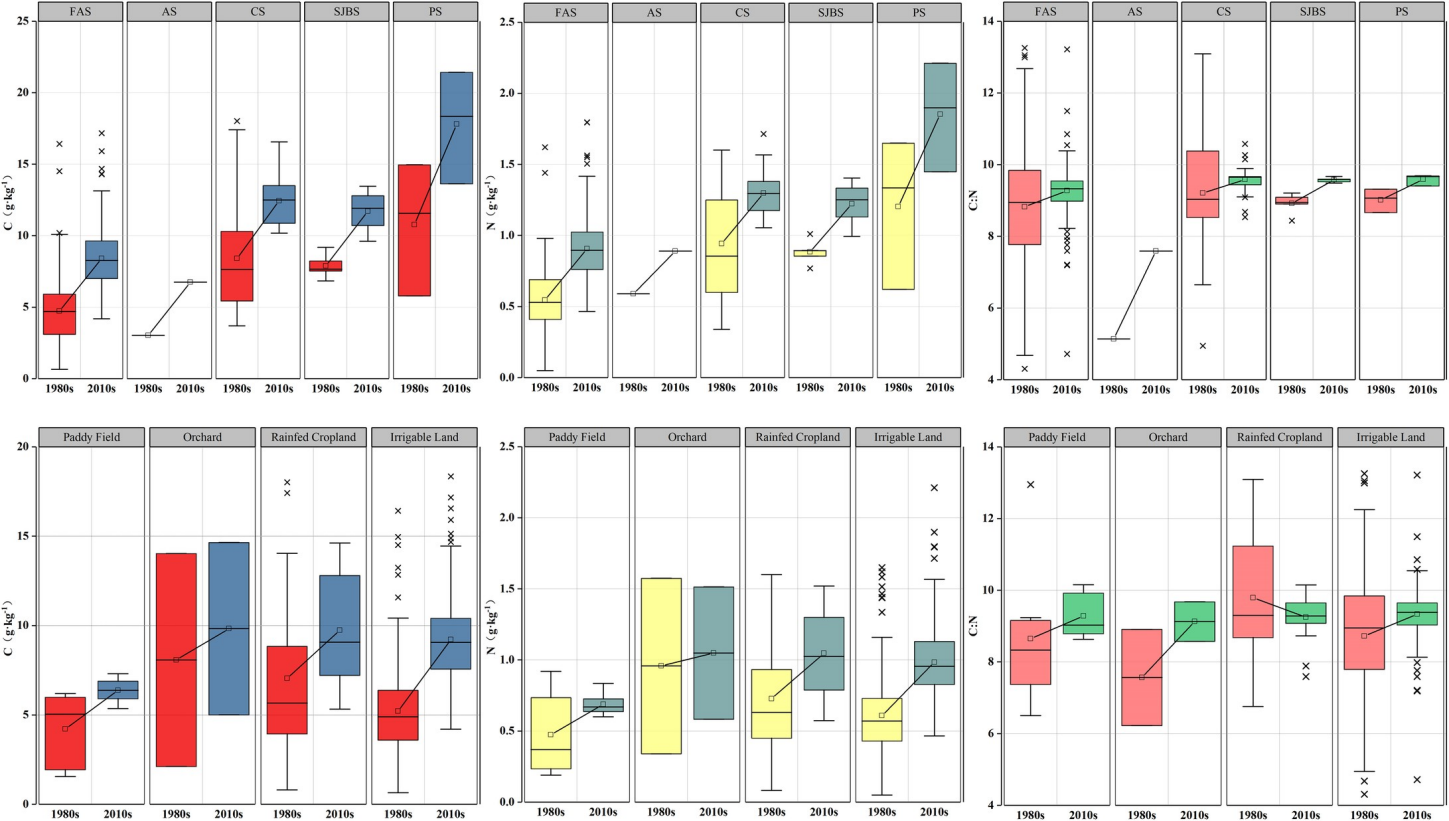
C, N contents and C:N stoichiometry in different soil types and agricultural land-use types (FAS: Fluvo-aquic soil, AS: Aeolian soil, CS: Cinnamon soil, SJBS: Shajiang black soil, PS: Paddy soil.).

The main soil types in the study area include fluvo-aquic soil, cinnamon soil, aeolian soil, shajiang black soil, and paddy soil. As Fig 4 shows, paddy soil had the highest C content, followed by cinnamon soil, shajiang black soil, fluvo-aquic soil and then aeolian soil in both the 1980s and the 2010s. Soil N content of different soil types was in sequence of paddy soil > cinnamon soil > shajiang black soil > aeolian soil > fluvo-aquic soil in the 1980s. In the 2010s, N content of fluvo-aquic soil exceeded that of aeolian soil. The significant test suggested that soil types have significant effects on C, N contents in both periods (Table 4). Paddy soil usually has the highest C, N contents because the parent materials are alluvial deposits and sediments, long terms of waterlogging induced bad permeability of the moisture, moreover, low temperature and inactive microbial activity slow down the decomposition of soil C and N, and facilitated nutrient accumulation [29]. Fluvo-aquic soil and aeolian soil had low C, N contents because these soil types are mostly composed of medium loam and sandy loam, their high porosity and poor water retention results in rapid decomposition of soil C and N [30].

**Table 4:** Significance test of soil types and land-use types on C, N contents and C:N stoichiometry.

Impact factors	Variable	F		P*	
		1980s	2010s	1980s	2010s
**Soil types**	C	20.155	43.576	.000	.000
	N	22.246	38.338	.000	.000
	C:N	.731	3.712	.571	.006
**Land-use types**	C	4.146	3.353	.007	.020
	N	2.690	3.657	.047	.013
	C:N	2.112	.183	.099	.908

*Significance level: *P* <0.05.

Additionally, we noticed that C:N ratio of aeolian soil was much smaller than that of other soil types in both two periods, which was similar to the result observed in other studies [5,31]. The possible reason for this phenomenon may be attributed to the unbalanced C, N accumulation of aeolian soils. On the one hand, short soil age and low development degree of aeolian soils resulted in less accumulation of soil organic carbon. On the other hand, long periods of intensive farming lead to a continuous increase of soil nitrogen [32,33].

### Effects of land-use types on soil C, N content and C:N ratio

Agricultural land-use types affect the input and output of soil nutrients through different accumulation and release rates of soil C and N, thus affecting soil C, N contents and C:N ratio. As Fig 4 shows, C content under different land-use types from high to low was as follows: orchard > rainfed cropland > irrigable land > paddy field in both periods. Soil N content of different land-use types in the 1980s was in the same order with C content, while in the 2010s, the sequence was rainfed cropland > orchard > irrigable land > paddy field. Table 4 indicated that land-use types also have significant effects on C, N contents in both the 1980s and the 2010s. The high C, N contents of the orchard and rainfed cropland might attribute to the large quantity of litterfall and less nutrient loss. Abundant litterfall of orchards provides plenty of nutrient elements into soils and resulted in high C, N contents of orchard soils [34]. The soil layer of rainfed cropland has good ventilation, which is beneficial to the microbial decomposition process, also, non-irrigation conditions reduced soil nutrient loss, and results in high contents of soil C, N in rainfed cropland [35]. The paddy field and irrigable land have low C, N contents because the humid soil environment enhances the reduction effects of soil microorganisms, and resulting in a decrease in soil nitrogen mineralization rate [36,37]. Furthermore, flood irrigation, which is widely applied in the North China plain made soil C, N more easily to be washed away along with surface runoff [38]. Soil C:N ratios of rainfed cropland and paddy field were higher than that of other land-use types, but the spatial difference between agricultural land-use types is not significant, this may be related to the fact that the research scale of this study is not large enough.

### Effects of topography on soil C, N contents and C:N ratio

**Fig 5:**
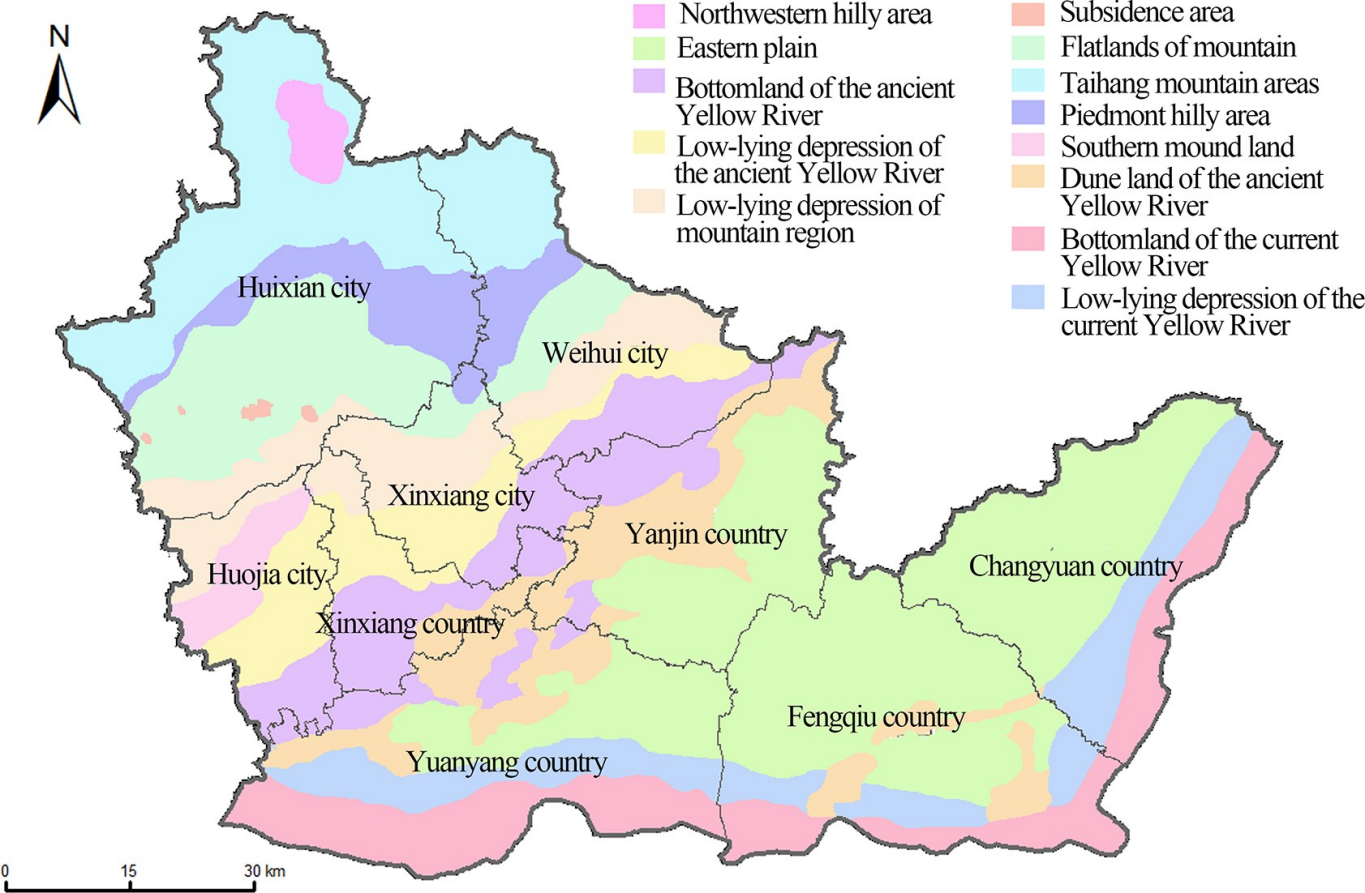
The geomorphic units of the study area.

The topography of the study area is varied and complicated (Fig 5), there were significant differences in soil C, N contents and C:N ratio between different geomorphologic units (Table 5). We observed that the high-value regions of soil C, N contents and C:N ratio were consistent with the location of Taihang mountain and piedmont hilly area, where the soil layer was thick and had better conditions for nutrient accumulation. C input was larger in the mountain area since it is dominated by forest and grassland, meanwhile, less human interference reduced the input of N. The differences in C, N inputs resulting in a high C:N ratio in the mountain and hilly regions. The low-value areas of soil C, N contents were mainly distributed in the "northeast-southwest" belt in Yanjin and Yuanyang counties, where the landform types were mainly bottomland and dune land of the ancient Yellow River. Due to the flood and erosion of the Yellow River in the historical period, soil in these areas has poor soil fertility and water retention ability, which may result in low soil C, N contents [39–41]. Furthermore, since these bottomland areas are suitable for agricultural cultivation, large nitrogen input in the agricultural production process results in a lower C:N ratio.

**Table 5:** Soil C, N contents and C:N ratio in different geomorphologic units.

Geomorphic units	C (g·kg^-1^)		N(g·kg^-1^)		C:N	
	1980s	2010s	1980s	2010s	1980s	2010s
Low-lying depression of the ancient Yellow River	5.36	9.77	0.57	1.04	9.47	9.36
Bottomland of the ancient Yellow River	4.99	9.21	0.58	1.01	8.77	9.18
Low-lying depression of the current Yellow River	4.31	7.29	0.50	0.79	9.05	9.27
Dune land of the ancient Yellow River	2.94	6.43	0.40	0.70	7.79	9.21
Bottomland of the current Yellow River	4.62	6.82	0.56	0.73	8.72	9.42
Northwestern hilly area	8.58	13.26	1.23	1.37	7.43	9.65
Piedmont hilly area	8.27	12.11	0.89	1.27	9.52	9.56
Subsidence area	6.84	9.97	0.74	1.05	9.27	9.48
Taihang mountain areas	11.28	13.07	1.19	1.35	9.86	9.67
Low-lying depression of mountain region	8.29	12.46	0.84	1.32	10.01	9.45
Flatlands of mountain region	7.52	12.04	0.82	1.26	9.29	9.58
Southern mound land	5.74	9.54	0.56	1.01	10.38	9.48
Eastern plain	3.90	7.61	0.50	0.83	8.22	9.21

### Effects of climate change and field management measures on soil C, N contents and C:N ratio

**Fig 6:**
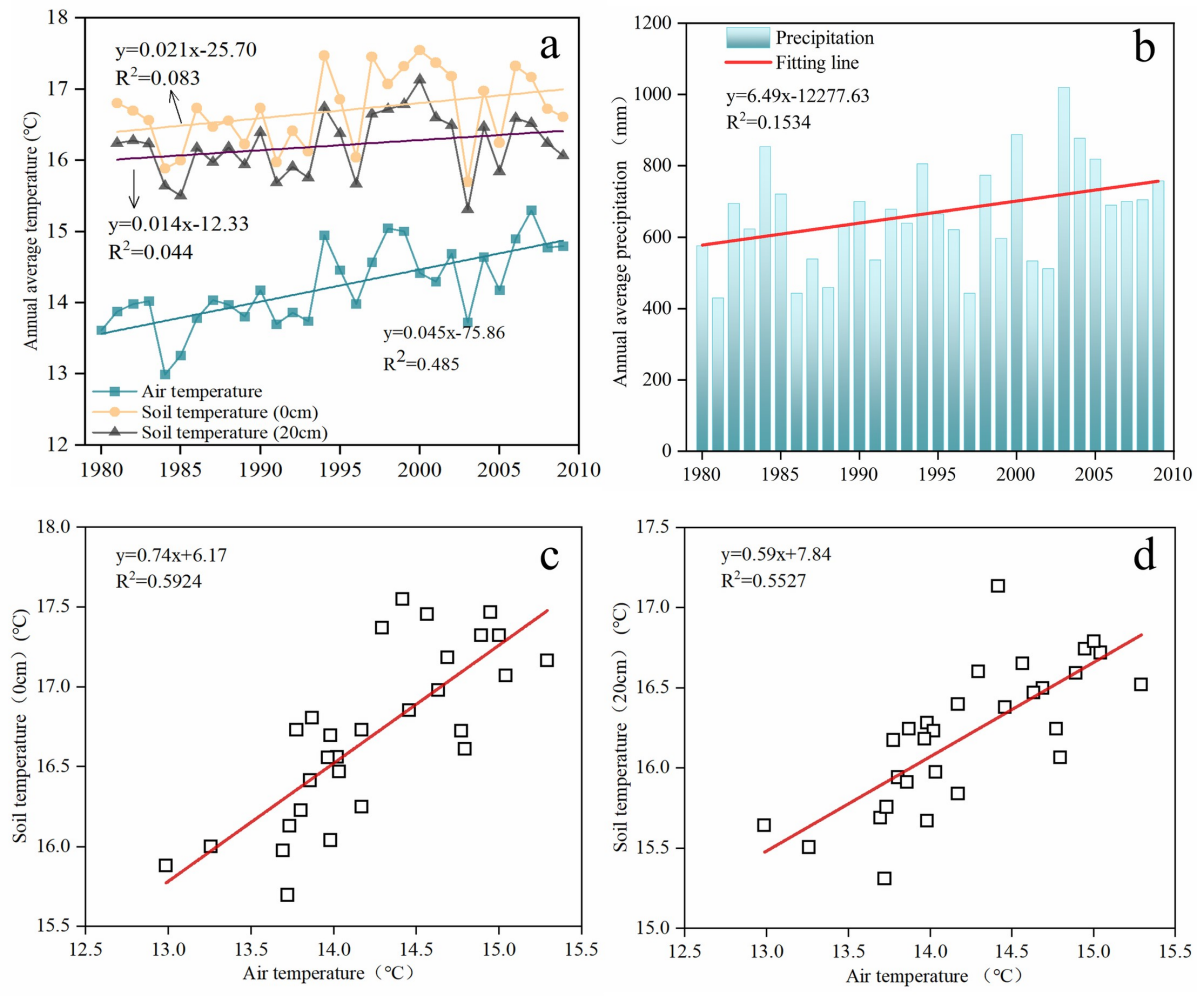
The climatic factors of the study area. a. Interannual variation of air temperature and soil temperature. b. Interannual variation of precipitation. c.d. Correlation between air temperature and soil temperature in the study area.

The annual average temperature in the study area increased significantly from 1981 to 2010, with a rate of 0.45°C every 10 years (Fig 6A), and there is a positive correlation between atmospheric temperature and soil temperature (Fig 6C and 6D). Fig 6B shows that there is a fluctuating uprising tendency of the average annual precipitation. Previous studies have suggested that climate change has profound impacts on temporal variation of soil C, N stoichiometry [42,43]. It is considered that the increased temperature speeds up soil drought, and slows down the decomposition rate and mineralization rate of soil C, N [44]. Meanwhile, the uprising tendency of precipitation results in increasing soil moisture, the moist soil environment may accelerate the mineralization rate of soil N, and consequently affects soil C:N ratio [45]. However, there are still uncertainties about the response of soil C, N stoichiometry to climate change, further study is needed to make quantitative evaluations of the relationship between climate change and stoichiometric characteristics of soil C, N stoichiometry.

**Fig 7:**
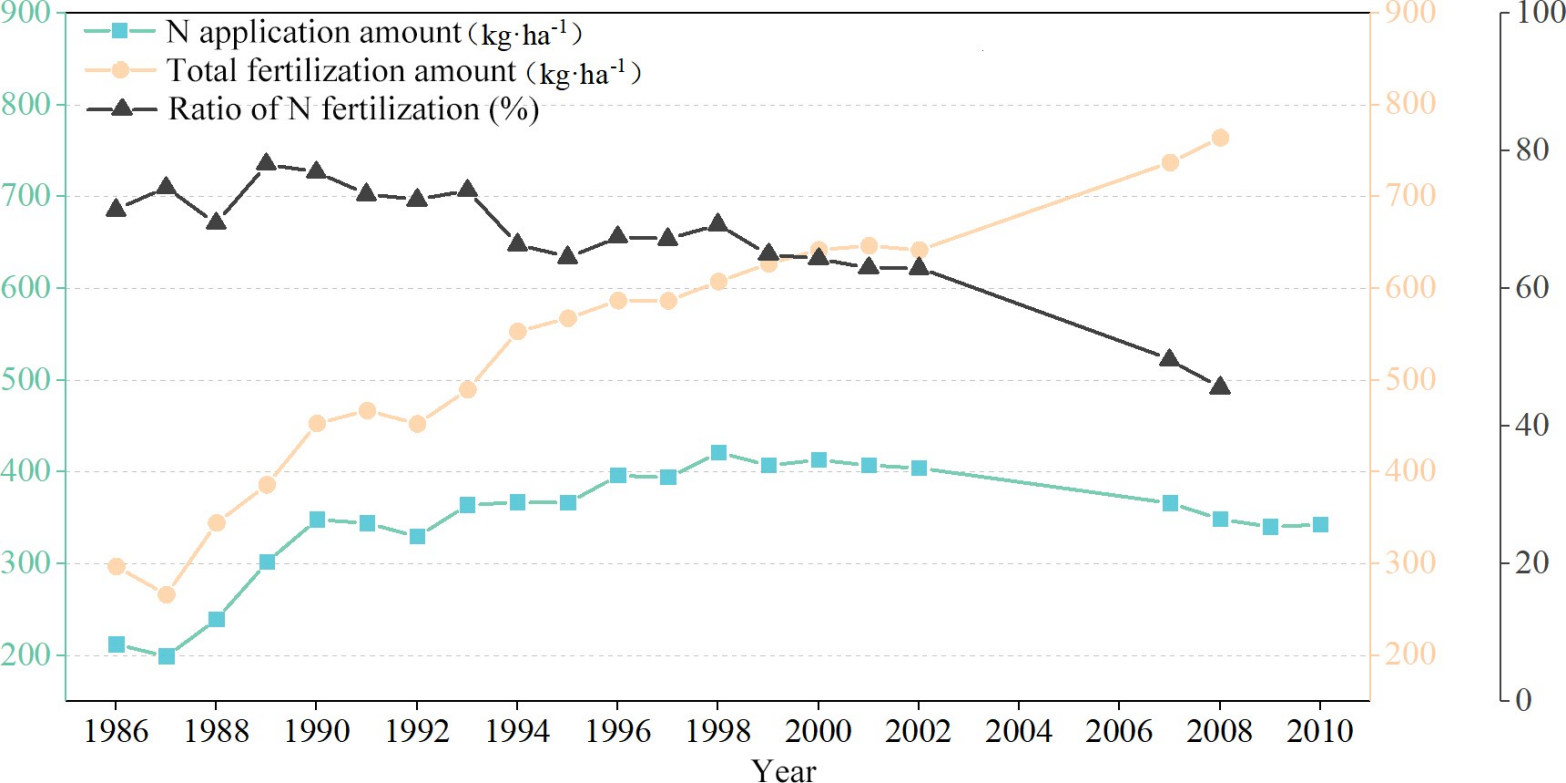
Interannual variation of fertilization amount.

Field management measure is another important influencing factor of soil C, N stoichiometry. Fig 7 suggested that the total amount of fertilizer applied per unit area in the study area showed an obvious increasing trend from the 1980s to the 2010s, but the ratio of N fertilizer was declining. Since the 21st century, soil testing and formulated fertilization technology were implemented and the straw returning measure was promoted, the proportion of nitrogen fertilizer decreased to 45.57%, and the fertilization structure was improved. In 2005, the Chinese government proposed the guideline of “promoting the comprehensive utilization and harmless treatment of organic fertilizers, guiding farmers to apply farmyard manure”, which provides strong policy orientation for field management. Previous studies have illustrated the application of organic fertilizer and straw incorporation could significantly increase the contents of soil C and N, as well as reduce the leaching of soil nutrients [46,47]. With the improvement of field fertilization and management methods, the nutritional status has shown some improvement in the major grain-producing region of the North China Plain, and this trend of improvement might be even more pronounced over a longer period.

## Conclusions

This study presented a temporal and spatial scale investigation on soil C:N stoichiometry in the major grain-producing region of the North China Plain. Soil C, N contents and C:N ratio have increased in varying degrees from the 1980s to the 2010s in the study area, but that was still lower than the contemporaneous national average value. Spatially, soil C, N contents changed from moderate spatial autocorrelation to strong spatial autocorrelation, the effect of random variation was reduced. Regions with high C, N contents and C:N ratio were mainly distributed in Huixian City and Weihui City, and the low-value area was concentrated in Yanjin County. During the past 30 years, C:N ratio has increased in most regions of the study area, it is conducive to the accumulation of soil organic carbon and the sustainable development of agriculture. C, N contents and C:N ratio showed respective variability characteristics under different soil types, agricultural land-use types, and topography. Climate change and field management measures were considered to be predominant factors of the temporal variation of soil C:N stoichiometry.

## Supporting information

S1 Table Soil profile data in the 1980s and the 2010s. (XLSX)
